# Prevalence, Characteristics, Association Factors of and Management Strategies for Low Back Pain Among Italian Amateur Cyclists: an Observational Cross-Sectional Study

**DOI:** 10.1186/s40798-021-00370-2

**Published:** 2021-10-28

**Authors:** Simone Battista, Lucia Grazia Sansone, Marco Testa

**Affiliations:** Department of Neurosciences, Rehabilitation, Ophthalmology, Genetics, Maternal and Child Health, University of Genova, Campus of Savona, Via Magliotto 2, 17100 Savona, SV Italy

**Keywords:** Low back pain, Prevalence, Cycling, Amateur cyclist, Rehabilitation, Disease management, Physical therapy modalities, Physiotherapy, Sports, Sports medicine

## Abstract

**Background:**

Low back pain (LBP) is a burdensome problem affecting amateur cyclists. This cross-sectional study analysed Italian amateur cycling cohort’s demographic and sport-specific characteristics, the prevalence and characteristics of LBP among this population, its possible association factors, the management strategies adopted to deal with LBP and the sample’s beliefs among possible LBP triggers. A web-based cross-sectional survey was created. The questionnaire included 56 questions divided into six sections, querying the sample’s demographic, clinical, and cycling characteristics. Binomial logistic regression with a Wald backward method was performed to ascertain the effects of some covariates (“Sex”, “Age”, “Body Mass Index”, “Sleep hours”, “Work type”, “Cycling year”, “Number of training sessions per week”, “Stretching sessions”, “Being supervised by a coach or following a scheduled training”, “Other sports practised regularly”, “Number of cycling competitions per year”, “Past biomechanic visits”, “Specific pedal training”, “LBP before cycling”) on the likelihood of developing LBP in the last 12 months.

**Results:**

A total of 1274 amateur cyclists answered the survey. The prevalence of LBP appeared to be 55.1%, 26.5% and 10.8% in life, in the last 12 months and the last 4 weeks, respectively. The final model of the logistic regression included the covariates “Sex”, “Work type”, “Cycling year”, “Being supervised by a coach or following a scheduled training”, “Other sports practised regularly”, “Specific pedal training”, “LBP before cycling”, among which “Cycling year” (variable “Between 2 and 5 years” vs. “Less than 2 years”, OR 0.48, 95% CI [0.26–0.89]), “Being supervised by a coach or following a scheduled training” (OR 0.53, 95% CI [0.37–0.74]), “Specific pedal training” (OR 0.69, 95% CI [0.51–0.94]), and “LBP before cycling” (OR 4.2, 95% CI [3.21–5.40]) were found to be significant.

**Conclusions:**

The prevalence of LBP among Italian amateur cyclists seems to be less frequent compared to the general population. Moreover, undergoing previous specific pedal training and being supervised by a coach or following scheduled training drew a negative association with LBP development. This evidence highlights the importance of being overseen by specific sport figures that could offer a tailored evidence-based training to reach good physical level and to practise sports safely.

**Supplementary Information:**

The online version contains supplementary material available at 10.1186/s40798-021-00370-2.

## Key points

- The prevalence of LBP among Italian amateur cyclists seems to be less frequent compared to the general population
- Following specific training (e.g. pedal training), being supervised by a coach or following structured training seems to be negatively associated with LBP development
- Amateur cyclists should be overseen by specific sport figures that can provide tailored and evidence-based training to practise sports safely

## Introduction

In the last few years, cycling has raised policymakers’ interest, due to the reported beneficial effects it has on both people’s health [1] and on the environment [2]. Although cycling can be considered as a low-impact activity, cyclists experience a broad array of musculoskeletal conditions such as patellofemoral pain syndrome, iliotibial band syndrome, Achilles tendinopathy, neck pain and low back pain [3, 4]. Limited flexibility, strength, and muscular recruitment patterns seem to be possible risk factors related to these conditions [5, 6].

In particular, low back pain (LBP) is a common and burdensome problem for cyclists. Evidence suggests that this can be due to the fact that prolonged postures are needed to achieve proper aerodynamics aimed at increasing speed and efficiency, including maintained lumbar spine flexion, which is associated with LBP [7, 8]. Moreover, the flexion-relaxation phenomenon, overactivation of the erector spinae, mechanical creep, and generation of high mechanical loads while being flexed and rotated are considered risk factors for LBP genesis in this population [8].

The quality of the equipment significantly affects cycling performance and efficiency [9, 10] since inadequate bike equipment may contribute to pain and injury [9, 11]. For instance, regulating the handlebar height correctly seems to prevent LBP, as a shallow handlebar position or riding with the hands gripping the lowest part of the handlebars in the so-called drop position contributes to LBP in cyclists [3, 9].

As for amateur cyclists, the prevalence of LBP over a long period was measured between 30 and 50%, dropping at 3–16% if measured over a short period (6—8 days) [12, 13]. Conversely, the prevalence of back pain is found to be between 58 and 60% among professional cyclists, as they are more exposed to fatigue, body overuse and psychological arousal [14, 15].

However, the data collected so far on amateur cyclists are not easily interpretable, due to the lack of standards for defining LBP, the small sample sizes analysed so far, and the different time frame in which the disease was investigated. Health professionals and physicians, who intend to become involved in the treatment of LBP in cyclists, should be aware of the plethora of issues related to this sport when assessing an injured amateur cyclist, the management strategies adopted, as well as of the possible association factors linked to LBP. In line with this, the primary aims of this study are (1) to depict an Italian amateur cycling cohort’s demographic and (2) sport-specific characteristics, (3) analyse the prevalence, and the characteristics of LBP among this sample, as well as its onset during cycling activities, (4) the management strategies (i.e. self-management strategies, use of medications, and the type of health professional they visit) used to deal with it and the (5) possible association factors related to this pathology. Besides, the secondary aim is to (6) understand the sample’s beliefs among possible LBP triggers related to the cycling activities (e.g. set-up of the bicycle).

## Methods

### Study Design

A quantitative web-based cross-sectional survey was developed according to the *International Handbook of Survey Methodology* through distinct and iterative steps to study Italian amateur cycling cohort’s demographic and sport-specific characteristics, the prevalence and characteristics of LBP among this population, as well as its onset during the cycling activities, its possible association factors, its management and sample’s beliefs among possible LBP triggers (Additional file 1: Appendix—translated version of the questionnaire) [16]. The study was conducted following the Declaration of Helsinki. Ethical approval was obtained from the Ethics Committee for University Research (CERA: Comitato Etico per la Ricerca di Ateneo), University of Genova (approval date: 25/06/2020; CERA2020.13) and follows the Strengthening the Reporting of Observational Studies in Epidemiology (STROBE) recommendations for reporting observational studies [17].

### Survey Development

A pool of physiotherapists developed the questionnaire. Throughout the questionnaire, the definition of LBP adopted followed the guidelines for a standardised LBP definition in the prevalence studies [18]. The questionnaire included a total of 56 questions, some of which could be bypassed according to previous answers, following a predetermined logic. The questions were divided into six sections. *Questions 1–3* (1st section—exclusion criteria) identified any exclusion criteria, in particular having vertebral fractures or back surgery, severe spondylolisthesis (3rd degree or greater), or suffering from any rheumatic disease. *Questions 4–10* (2nd section—Descriptive analysis) investigated the characteristics of the participants (sex, age, height, weight) and their lifestyle habits (smoking, hours of sleep per night, occupation). *Question 11–24* (3rd section—Cycling-related questions) analysed the participants’ cycling activity (years of cycling, memberships), their workout (number/hours of workout per week, specific types of training followed, use of activity trackers, number and type of races, if they were regularly practising other sports apart from cycling and/or stretching sessions and if they were supervised by a coach or if they were followed a scheduled training) some technical aspects regarding the pedalling technique (specific training for pedalling technique), and the bicycle set-up (questions about previous biomechanical visits). *Questions 25–37* (4th section—Prevalence of LBP) investigated the prevalence of LBP in life, in the 12 months, in the last 4 weeks and the characteristics of the last episode of back pain (intensity and irradiations) and if sciatica, defines as “Pain radiating down the leg below the knee in the distribution of the sciatic nerve” [19], was present. *Questions 37–50* (5th section—LBP and cycling) examined the presence or absence of LBP during cycling, the characteristics of this pain, the factors that participants held responsible for its origin, and their beliefs concerning any correlation between LBP and their cycling activity. *Questions 51–56* (6th section—LBP management) examined the participants’ behaviours to manage their LBP.

### Participants

This online questionnaire addressed amateur cyclists, i.e. people who had been practising cycling as a sport for more than one year, either at a competitive or non-competitive level, but not at a professional level. Participants were not informed in advance on the fact that the focus of the questionnaire was LBP, since cyclists who experienced this disease could have been more prone to partake in this questionnaire compared to their counterpart without LBP. Participants were informed in advance about the time required to complete the questionnaire, as well as of the anonymity of the information collected in the survey. Amateur cyclists were reached through instant messaging platforms, social network websites and mailing lists of cycling companies previously contacted by telephone. The individual’s decision to fill in the online questionnaire was spontaneous, and there was no incentive. The data were collected from 15 to 30 July 2020.

### Analysis

For the analysis of the results, all the incomplete questionnaires were excluded. Descriptive analysis was carried out in order to understand the sample’s characteristics. Continuous variables were reported as mean ± standard deviation (SD), while categorical variables were reported as absolute and percentage frequencies. A prediction model with a logistic regression—Wald backward method—was performed to evaluate the impact of the covariates “Sex” (dichotomous variable: female/male) “Age” (continuous variable), “Body mass index” (continuos variable), “Sleep hours” (categorical variable: less than 6 h/between 6 and 7 h/between 7 and 8 h/more than 8 h), “Work type” (categorical variable: sedentary/statistic/dynamic/heave/unemployed), “Cycling year” (categorical variable: less than 2 year/between 2 and 5 years/more than 5 years), “Number of training per week” (once to twice, thrice to four times, five to six times, more than six times), “Stretching sessions” (categorical variable: never, rarely, once or twice a week, often), “Being supervised by a coach or following a scheduled training” (dichotomous variable: yes/no), “Other sports practised regularly” (dichotomous variable: yes/no), “Number of cycling competitions per year” (categorical variable: between 1 and 4, between 5 and 8, between 9 and 12, more than 12, none), “Past biomechanic visit” (dichotomous variable: yes/no), “Specific training pedal” (dichotomous variable: yes/no), “LBP before cycling” (dichotomous variable: yes/no), on the likelihood of developing LBP in the last 12 months (dichotomous variable: yes/no). The final model included the covariates “Sex”, “Work type”, “Cycling year”, “Being supervised by a coach or following a scheduled training”, “Other sports practised regularly”, “Specific training pedal”, “LBP before cycling”. Instead, the variables “Age”, “Body mass index”, “Sleep hours”, “Number of training per week”, “Stretching sessions”, “Number of cycling competitions per year”, “Past biomechanic visit” (dichotomous variable: yes/no) were deleted from the final model. The linearity of the continuous variables for the logit of the dependent variable was assessed via the Box–Tidwell procedure. A Bonferroni correction was applied using all twenty terms in the model, resulting in statistical significance being accepted when *p* < 0.01. Based on this assessment, all continuous independent variables were found to be linearly related to the logit of the dependent variable. There was no standardised residual assessing in the casewise list. Odds ratio (OR) and 95% CI were estimated for each covariate reference category.

## Results

### Participants

**Fig. 1:**
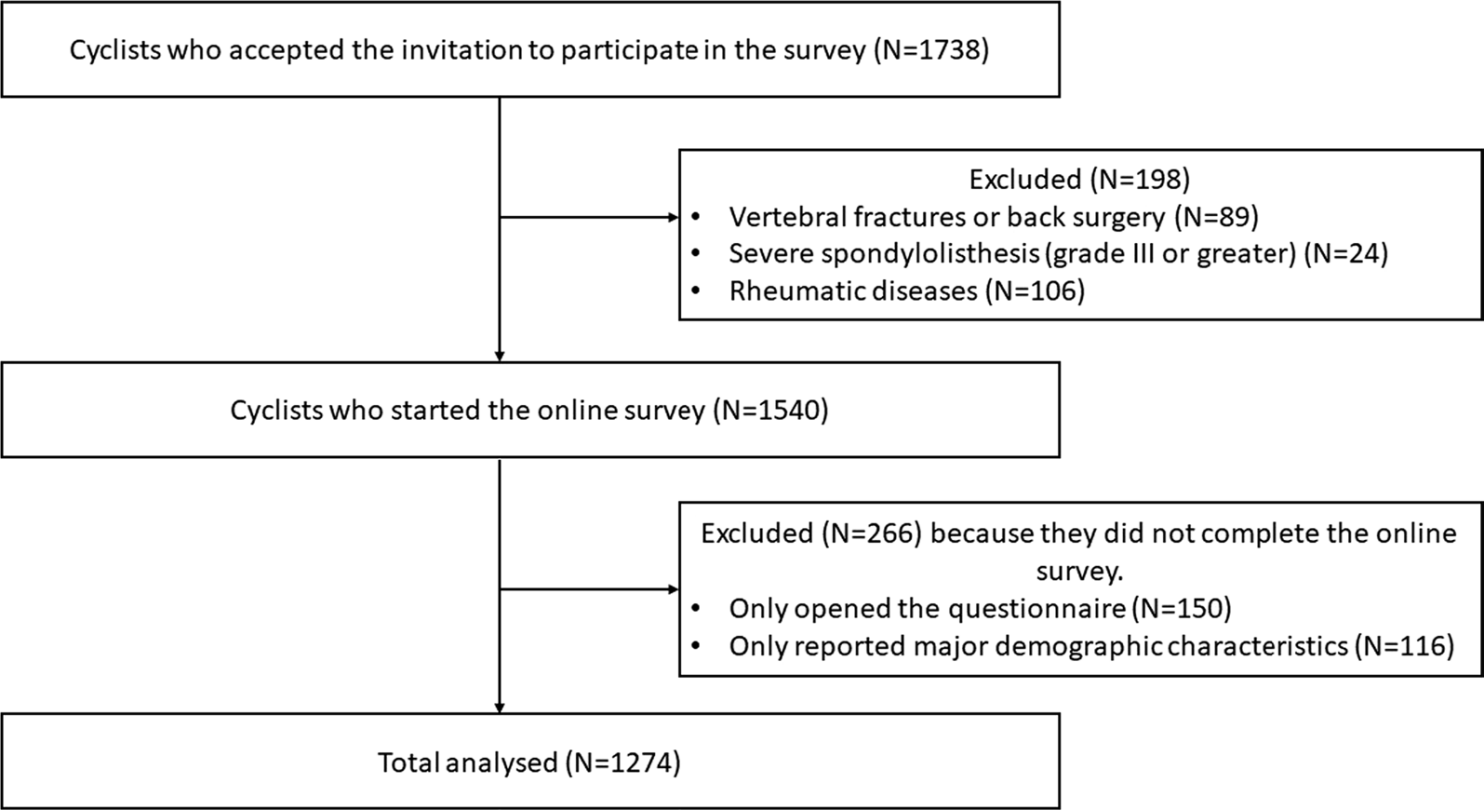
Participants’ flowchart

The questionnaire was sent to 1738 people. Of these, 1274 (response rate: 73.3%) completed the questionnaire entirely, 266 (15.3%) did not complete it or did not answer it. Among these, 150 (8.6%) only opened the questionnaire and 116 (6.7%) only reported the major demographic characteristics (sex, age and BMI) and exited the questionnaire. These did not differ significantly as far as sex (*X*^2^ = 0.225, *p* > 0.05), age (*z* = − 0.47, *p* > 0.05) and BMI (*z* = − 0.751, *p* > 0.05) are concerned when compared to those who completed the whole questionnaire. Moreover, 198 (11.4%) were excluded because of one or more exclusion criteria. Specifically: 89 people (5.1%) had vertebral fractures or back surgery, 23 people (1.3%) had severe spondylolisthesis (grade III or greater), and 106 people (6.1%) suffered from rheumatic diseases. The average time to complete the entire questionnaire was about 11 min. Thus, 1274 (mean age (SD): 43.18 (12.21); women 11%; men 89%) amateur cyclists completed the questionnaire in all its sections and were included in the analysis (Table 1, Fig. 1).

**Table 1:** Participants’ demographic and lifestyle characteristics

	*N* = 1274
Age (years) (mean, (SD))	43.18 ± 12.21
Sex (female, male) (*N* (%)):	146 (11.5), 1128 (88.5)
Body Mass Index (mean, (SD))	23.20 ± 2.80
Sleep hour (*N* (%))	
Less than 6 h	107 (8.4)
Between 6 and 7 h	598 (46.9)
Between 7 and 8 h	476 (37.4)
More than 8 h	93 (7.3)
Smoke (yes, no) (*N*%)	76 (6.0), 1198 (94.0)
Work-type degrees (*N* (%))	
Sedentary	571 (44.8)
Static	55 (4.3)
Dynamic	383 (30.1)
Heavy	124 (9.7)
Unemployed	141 (11.1)

*N* number, *SD* standard deviation

### Analysis of the Sections of the Survey

#### Participants’ Cycling Characteristics

Table 2 reports all the sample’s characteristics concerned with the cycling activities, and Table 3 gives the information regarding the biomechanical visits attended.

**Table 2:** Participants’ cycling characteristics

	*N* = 1274; *N* (%)
Cycling year	
Less than 2 years	79 (6.2)
Between 2 and 5 years	247 (19.4)
More than 5 years	948 (74.4)
Membership	
Competitor	791 (62.1)
No competitor	110 (8.6)
No membership	373 (29.3)
Training per week	
Once to twice	402 (31.6)
Thrice to four times	599 (47.0)
Five to six times	227 (17.8)
More than six times	46 (3.6)
Training per hour	
Less than 5 h	267 (21.0)
Between 5 and 12 h	758 (59.5)
Between 13 and 20 h	210 (16.5)
More than 20 h	39 (3.0)
Being supervised by a coach or following a Scheduled training (yes, no)	335 (26.3), 939 (73.7)
Device assessment	
Heart rate	309 (24.3)
Heart rate + Power Monitor	644 (50.5)
None	321 (25.2)
Specific pedal training (yes, no)	820 (64.4), 454 (35.6)
Cycling competitions per year	
Between 1 and 4	361 (28.3)
Between 5 and 8	146 (11.5)
Between 9 and 12	98 (7.7)
More than 12	123 (9.7)
None	546 (42.8)
Type of competition^†^	
Track cycling	114 (15.6)
Cyclosportive	585 (80.3)
Hillclimbing	30 (4.1)
Stretching sessions	
Never	230 (18.1)
Rarely	553 (43.4)
Once or twice a week	287 (22.5)
Often	204 (16.0)
Other sports practised regularly (yes, no)	737 (57.8), 537 (42.2)

*N* number. ^†^*N* = 729

**Table 3:** Participants’ biomechanical visit

	*N* = 1274; *N* (%)
Biomechanical visit (yes, no)	691 (54.2), 583 (45.8)
Reason to attend a biomechanical visit^†^	
Improve performance	233 (33.7)
Injury prevention	200 (28.9)
Pain/discomfort	258 (37.4)
Pain/Discomfort after biomechanical visit^‡^	
Unchanged	9 (3.5)
Partially improve	81 (31.4)
Sharply improved	100 (38.8)
Completely disappeared	68 (26.3)

*N* number. ^†^*N* = 691; ^‡^*N* = 258

#### LBP Prevalence

**Fig. 2:**
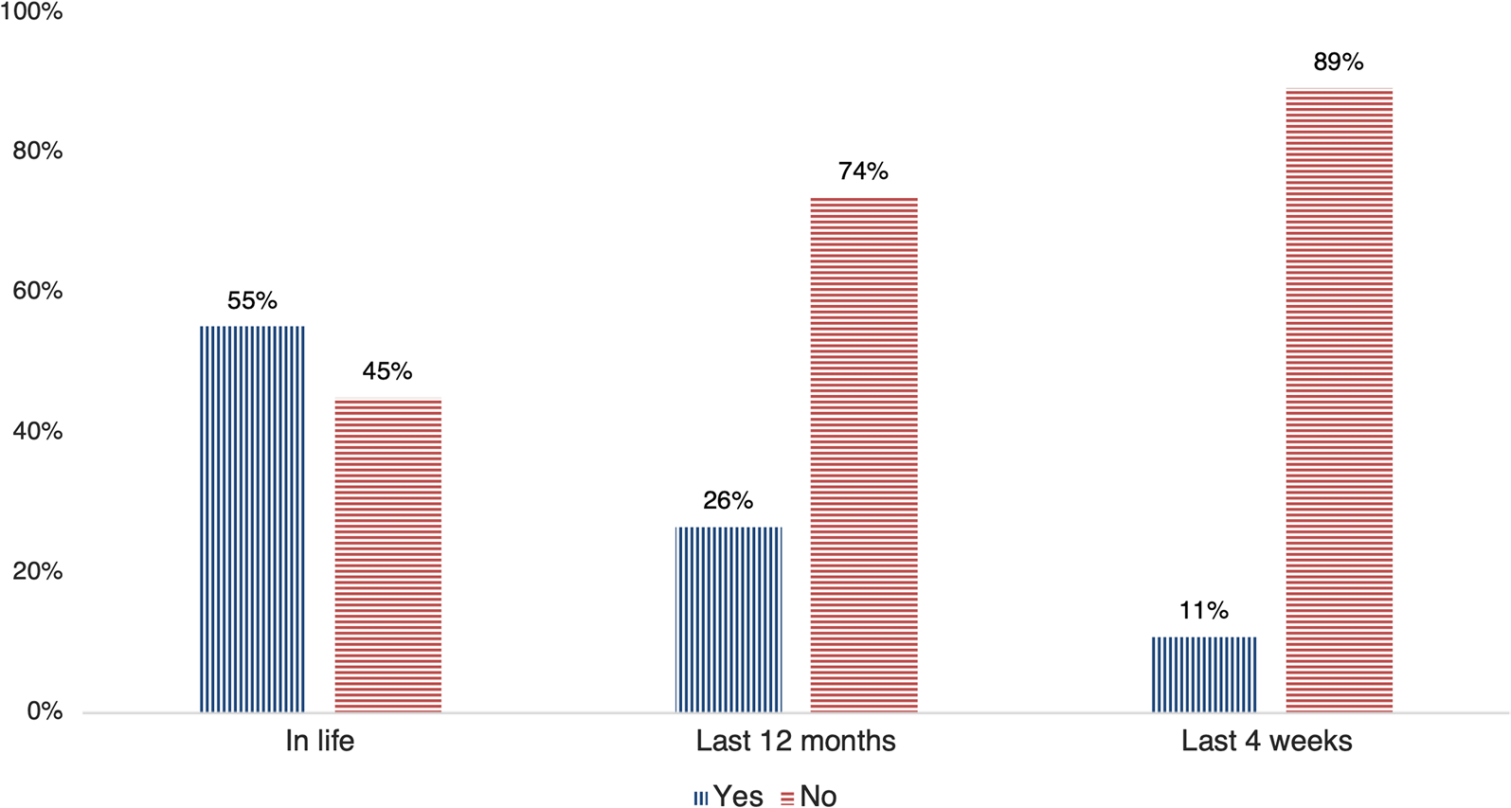
Low back pain prevalence among Italian amateur cyclists

**Fig. 3:**
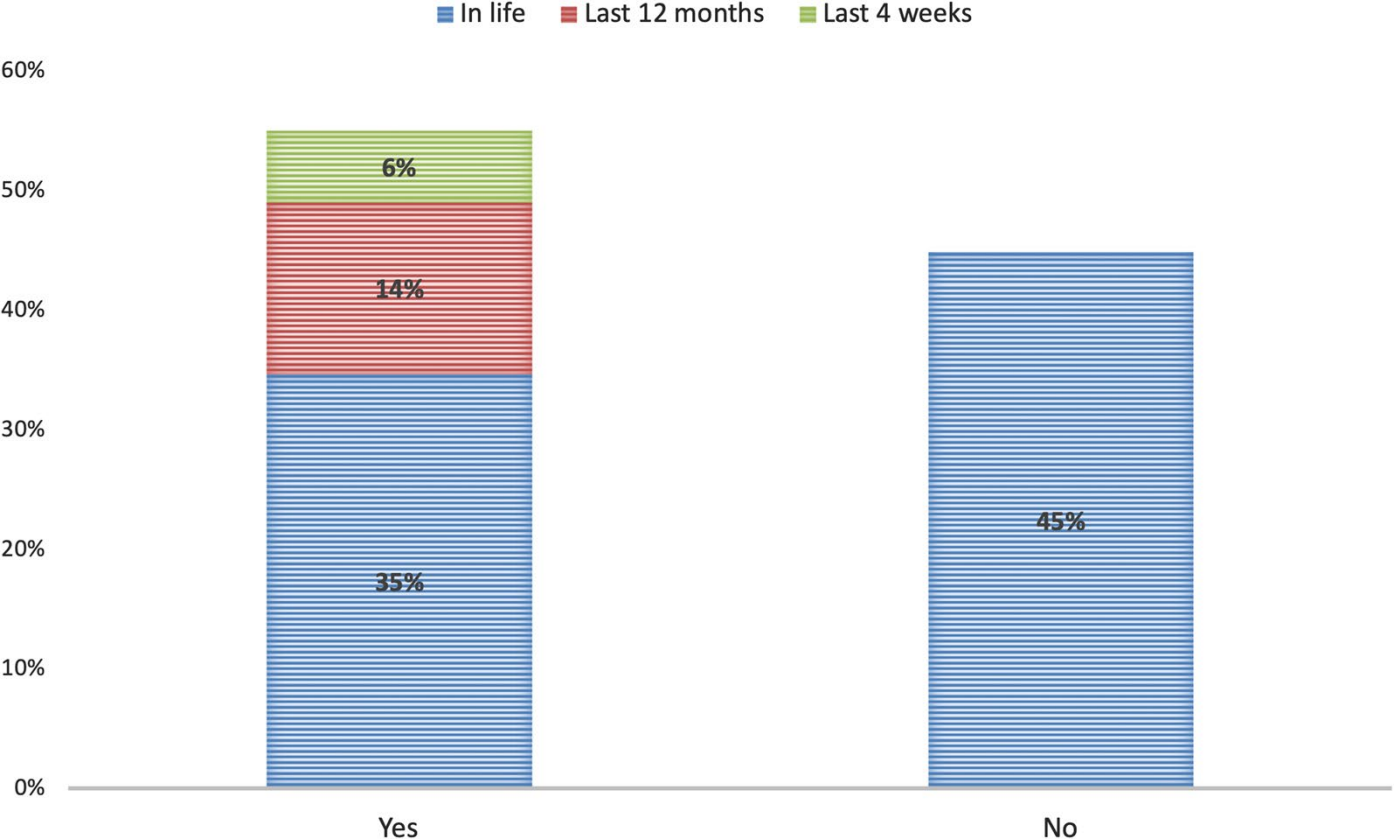
Low back pain cumulative prevalence percentage among Italian amateur cyclists

Table 4 and Fig. 2 report the prevalence of LBP in life, in the last 12 months, and in the last 4 weeks and the symptoms’ characteristics. Precisely, 55.1%, 26.5% and 10.8% reported suffering from LBP in life, in the 12 months and the last 4 weeks, respectively (Fig. 2). Since the participants that experienced pain in the last 4 weeks answered positively to having had LBP in the last 12 months and in life, and the participants that experienced pain in the last 12 months answered positively to having had LBP in life, the cumulative percentage for the three categories of LBP are also reported in Fig. 3. In particular, 35%, 14% and 6% reported suffering from LBP in life, in the 12 months and the last 4 weeks, exclusively, whereas 45% reported not to have suffered from LBP in their life.

**Table 4:** Prevalence of LBP and symptoms characteristics

	*N* = 1274; *N* (%)
LBP in life	702 (55.1)
Number of episodes (mean ± SD)^†^	6.6 ± 10.24
Pain radiating to the lower limbs^†^	326 (46.4)
To the knee	191 (58.6)
Beneath the knee	135 (41.4)
Other symptoms to the lower limbs	326 (25.6)
Type of the other symptoms to the lower limbs^‡^	
No (just pain)	113 (34.7)
Tingling	143 (43.9)
Burning	56 (17.2)
Strength loss	67 (20.6)
Sensitivity loss	60 (18.4)
LBP in the last 12 month	337 (26.5)
Number of episodes (mean ± SD)§	3.6 ± 7.8
LBP in the last 4 weeks	137 (10.8)
Number of episodes (mean ± SD)^¶^	2.8 ± 8.8

*N* number, *SD* standard deviation. ^†^*N* = 702; ^‡^*N* = 326—multiple choice allowed; ^§^*N* = 337; ^¶^*N* = 137

Finally, Table 5 reports the characteristics of the last LBP episode and the presence of sciatica during this episode.

**Table 5:** Characteristics of the last LBP episode

	*N* = 702; *N*(%)
Pain duration	
Some days	564 (80.4)
One month	73 (10.4)
Three months	31 (4.4)
More than 3 months	34 (4.8)
Pain intensity (NRS) Q2 [Q1, Q3]	5 [4–7]
Pain radiating to the lower limbs	192 (27.4)
Pain coinciding with a period of stress	262 (37.3)

*N* number, *NRS* numeric rating scale, *Q2* second quartile, *Q1* first quartile, *Q3* third quartile

#### LBP Onset and Cycling Activities

In our sample, 581 people (45.6%) reported suffering from LBP at least once in their life during or after a cycling session. In 32.1% of cases, LBP was presented only under load, in 54.9% of cases, it appeared after a few hours no matter the effort was, and in 13% of cases, it appeared immediately before the effort. Moreover, 150 people (25.8%) had never suffered from LBP before starting cycling, whereas 431 (74.2%) had already suffered from LBP before starting cycling. In our cohort of participants, 71.6% of cyclists reported that LBP did not coincide with a particular period of the season, 17.4% said that it coincided with a training period, 6.5% suffered from LBP during the competition period, 3% during the pre-competition period and 1.5% during the rest period. Among those who replied that LBP on bicycles coincided with a particular training period, the primary type of training to which cyclists were subjected at that time was investigated. In particular, 32.6% were riding long climbs (20–40 min) at their anaerobic threshold intensity, 22.7% were performing strength exercises with a low pedalling cadence (Resistant Strength Climb), 14.2% were riding long climbs (> 30 min) but at low intensity climbs, in 14.2% cases, it was plain training at aerobic intensity, in 7.8%, it consisted of short repetitions (2–5 min) uphill at high intensity, in 5.7%, it consisted of long repetitions (10–15 min) uphill at high intensity, and in 2.8%, they were riding maximum shots lasting between 20 and 60 s.

#### LBP and Association Factors

A binomial logistic regression was performed to ascertain the effects of “Sex”, “Work type”, “Cycling Year”, “Being supervised by a coach or following a scheduled training”, “Other sports practised regularly”, “Specific Training Pedal”, “LBP before cycling” on the likelihood that participants had suffered from LBP in the last 12 months. The logistic regression model was statistically significant, *χ*^2^(4) = 159.81, *p* < 0.01. Of the abovementioned predictor variables, only five were statistically significant, as shown in Table 6, together with their OR.

**Table 6:** Logistic regression LBP 12 months

*N* = 1274	Sig	Odds ratio	95% CI for EXP(B)	
			Lower	Upper
Sex (female, male)	0.09	0.67	0.42	1.07
Work type (Unemployed—ref. category)	0.07			
Cycling year (< 2 years—ref. category)	< 0.01*			
Between 2 and 5 years	0.02*	0.48	0.26	0.89
More than 5 years	0.80	0.93	0.54	1.61
Being supervised by a coach or following a scheduled training (yes)	< 0.01*	0.53	0.37	0.74
Other sports practised regularly (yes)	0.08	1.28	0.97	1.69
Specific Pedal Training (yes)	0.02*	0.69	0.51	0.94
LBP Before Cycling (yes)	< 0.01*	4.20	3.21	5.40
Constant	< 0.01*	0.26		

*N* number, *NRS* numeric rating scale, *CI* confidence interval, *EXP(B)* exposure—LBP 12 months (NO/YES)

*Statistically significant

^†^The logistic regression model is based on a Wald backward stepwise model

^‡^The variables “Age”, “Body mass index”, “Sleep hours”, “Number of training per week”, “Stretching sessions”, “Number of cycling competitions per year”, “Past biomechanic visit” (dichotomous variable: yes/no) were deleted from the final model

#### LBP Management

The main strategies used by cyclists to reduce back pain while cycling were the following: to extend their back (54.4%), to reduce the speed of their pedalling or the intensity of the effort (11.8%), to flex their back (9.3%), to pedal faster (7.8%), to stop and get off the bike (4.7%) or to pedal harder (0.5%). However, in 11.5% of the presented cases, none of the previous strategies exposed reduced LBP while cycling. Finally, 249 participants (19.5%) reported that their LBP had affected their training somehow (suspension, decrease in intensity and/or frequency, variation in type).

Among the cyclists who have suffered from LBP in their life, 52.7% of them never turned to a professional figure to deal with their pain, whereas the remaining 47.3% did. Among these, the professional figures they turned to in order to manage their LBP were physiotherapists (63.3%), osteopaths (50.0%), physicians (38.1%), biomechanics (i.e. figures that assess and correct the right saddle position for cyclists; 30.8%), masseurs (25.5%) and chiropractors (12.2%).

Among the cyclists who suffered from LBP in their life, 35.8% took medications to reduce their pain at least once. The most common drugs used were non-steroidal anti-inflammatory drugs (NSAIDs; 79.3%), analgesic drugs (13.0%) and cortisone (7.7%). Among those who took drugs, 70.7% said that their physician had prescribed them, 4.5% said that friends or family recommended the drugs, and the remaining 27.8% said they had taken medicines on their initiative.

Finally, the participants who declared that they had suffered from back pain were asked if they had ever practised bodyweight exercises to strengthen their back: 22.2% of these had never practised this type of exercises, 29.0% practised them sporadically, 18.2% practised them only in specific periods of the year, 22.7% practised them once or twice a week, and 7.9% practised them almost every day.

#### Cyclists’ Beliefs Regarding LBP Onset

As far as the perceived correlation between LBP and equipment is concerned, 31.3% of the surveyed population stated that, while riding, LBP arose as a result of a change in the set-up of the bicycle (saddle height or backward, saddle-handlebar distance or difference, the position of the cleats) or a change of some component of the bike itself (saddle, handlebars, pedals).

Among the cyclists who outlined a possible correlation between LBP and cycling activity, 56.0% believed that cycling does not affect LBP in any way, 26.3% believed that cycling negatively affects LBP, and 17.7% believed that cycling positively affects LBP. Finally, 34.5% of those who, at least once in their lifetime, had suffered from LBP during or after cycling, thought that the position on the bicycle might be the cause of LBP.

## Discussion

As far as the prevalence of LBP among amateur cyclists is concerned, our study conveyed that 55.1%, 26.5% and 10.8% of amateur cyclists reported suffering from LBP in life, in the last 12 months and the last 4 weeks, respectively. In line with these results, amateur cyclists seem to have less frequent LBP than the general population, whose lifetime prevalence of non-specific (common) LBP is estimated to be between 60 and 70%, with a one-year prevalence between 15 and 45% and a 4 weeks prevalence of 30.8 [20–23]. It is well known that a good level of physical activity, compared to inactivity or low-level of activity, is able to reduce the risk of LBP and its associated disability [24, 25], especially if combined with aerobic exercises as in the case of cyclism [24]. However, as far as the intensity of pain and the presence of sciatica (10.5% of the entire sample), during the last LBP episode, are concerned, they were similarly experienced by our sample compared with the general population [26, 27].

As a matter of fact, the physical activity is not beneficial per se*,* since it needs to be tailored on the individual’s capacity, taking into account muscular tissues load resistance without overloading them. Consistent with this, when compared to our data, the annual prevalence reported by professional cyclists (58%) is significantly higher [28]. These results suggest that the physical and psychological loads to which professional cyclists are subjected could influence their annual prevalence of LBP. Unfortunately, there are no data on the lifetime and 1-month prevalence among professional cyclists. Therefore, it is not possible to draw a comparison between these two populations in the abovementioned timeline.

When compared to other sports, one notices that the prevalence of LBP in life among amateur cyclists is similar to what has been found among rowers (64.7%) [29]. These two sports are characterised by high training volumes and repetitive movements, like forward flexion of the trunk, which may be responsible for their similar prevalence rates [30]. Moreover, our data are also comparable to the data retrieved for football, handball, ice hockey, field hockey, basketball, and rugby. All these sports are characterised by high physical loads, repetitive mechanical strain, static and extreme postures, which all relate to the risk of LBP, whose prevalence was 1–64% [31]. Conversely, all these data contrast with the findings retrieved among runners, who reported a lower LBP prevalence, suggesting that running might be a protective factor for the development of LBP [23, 29]. However, these data must be considered cautiously because of the different LBP definitions adopted throughout all the abovementioned studies, the sample’s characteristics, and the wide prevalence ranges reported.

Training errors, such as the ones that may occur in the pedalling technique, seem to be major risk factors in cyclists when it comes to pathologies such as patellofemoral pain syndrome and Achilles tendinopathy [3, 4]. However, none of the previous researches investigate the relationship between pedalling technique and LBP. The present study is thus the first to bridge this knowledge gap in the LBP onset. Specifically, the performed logistic regression found that the covariates of “Specific pedal training” (OR 0.69) and “Being supervised by a coach or following a scheduled training” (OR 0.53) seem to be modest and moderate negative association factors, respectively.

Some hypotheses that may explain why pedalling training is a negative association factor for LBP are that a pedalling technique that provides a more uniform delivery of force throughout the pedalling cycle [32] contributes to reducing the peak of force in the propulsive phase, with two major consequences, that is to say, (1) a lower peak of muscle activation of the lower limbs and (2) the stabilising muscles of the lumbo-pelvic area, with a consequent lower risk of overload for these structures. Consequently, the lower activation of the lumbo-pelvic muscles reduces the possibility of strain and fatigue of these muscles which are known to produce important kinematic changes such as greater lumbar and thoracic flexion, greater thoracic and pelvic tilt, or greater hip adduction, that could lead to lumbar injuries [33].

As far as being supervised by a coach or following a training programme is concerned, these aspects could be a negative association factors for the LBP as they both involve a planning of training loads, both in terms of intensity, volumes and frequency. The progressiveness and adequacy of training loads are at the basis of the reduction in the risk of onset of LBP [34, 35]. Furthermore, a structured training programme includes different types of training stimuli, including high-intensity interval training exercises or strength training, but amateur cyclists that are not supervised by a coach or that do not follow a training programme may tend to perform mostly resistance training at low intensity for long durations. Conversely, the highest positive association factor for LBP development identified was suffering from LBP before starting to cycle (OR 4.20) which corroborates the existing literature that considers previous LBP episodes as a positive association factor for the onset of future LBP attacks [21]. However, these data must be taken into account cautiously, due to the design of this study, which can outline possible associations but not cause-effect correlations.

In our sample, 47.3% of the cyclists, who suffered from LBP in their life, turned to a professional figure (health professionals or not) to try to solve their disease. Our sample differs from the general population, among which only about 15–20% patients with LBP sought professional intervention [36]. However, since first-line interventions for LBP (i.e. exercise and education) are mainly delivered by professional figures such as physiotherapists, our data highlight the needs to foster the importance of being able to refer to such figures [37] with the aim of reducing medication use and the possibility of chronicisation.

As far as pharmacological therapy is concerned, 35.8% of the cyclists with LBP declared using pain relief medications.. These data stand in contrast with the what has been retrieved when looking at the general population and the elite athletes with LBP, among whom 69.4% and more than 50.0%, respectively, reported using pain relief medication [38, 39]. However, in our sample, the most common drugs used were NSAIDs (79.3%). This is in line with a study by Outram et al., stating that the majority of amateur cyclists reported regular use of NSAIDs in combination with caffeine [40]. However, international clinical practice guidelines (CPGs) recommend avoiding use of NSAIDs in favour of exercise and education as a first-line treatment for chronic pain and that NSAIDs should only be used as a complementary treatment for a short period of time and during the acute phase, in order to reduce the possible side effects of these medications [41–43]. Therefore, prioritising the use of NSAIDs over other first-line interventions may jeopardise the success of the therapeutic process. In line with that, future studies should investigate amateur cyclists’ awareness of the importance of the different treatments they should undergo in order to manage their disease and to understand the perceived role that each treatment may play in their care process.

Some limitations of this study need to be discussed. First of all, the cross-sectional nature of the study did not allow for an evaluation of the causative relationship between LBP and technical cycling characteristics. Moreover, possible recall biasses may have taken place since some of the information requested was retrospective. Thus, future prospective cohort studies with specific outcome measures evaluating the impact of the different musculoskeletal disorders from which this population seems to suffer (e.g. LBP, neck pain, lower limb injuries) are necessary [44–46]. Secondly, the low presence of female or smoker cyclists could have biassed the results of this study as far as these two covariates were concerned. Thirdly, it is not possible to be sure that our final sample is representative of the entire population of amateur cyclists, since there is no national register keeping track of the number of people who are performing this sport at this level. However, the high response rate reached (> 70%) reduces the possibility of a sampling bias.

## Conclusions

This study is the first one to investigate LBP among amateur cyclists starting from an evidence-based definition of LBP, and which deeply depicts the demographic, clinical and technical cycling characteristics of this population. The main results of this study show that amateur cyclists seem to be less subjected to LBP compared to the general population and that undergoing specific training (e.g. pedal training) and being overseen by a coach or following a scheduled training could reduce the possibility of developing LBP. These results bring to the forefront the importance of physical activity as a means to maintain a good level of physical health and its preventive role in the development of pathological conditions, such as LBP. Nevertheless, our results also highlight the fact that physical activity should be individualised and bespoke to one’s needs and capacity in order to set adequate goals and avoid overuse injuries.

## Supplementary Information

**Additional file 1**. Translated version of the questionnaire provided to Italian amateur cyclists.

## Data Availability

The datasets used and analysed during the current study are available from the corresponding author on reasonable request.

## Abbreviations

LBP: Low back pain
NSAIDs: Non-steroidal anti-inflammatory drugs
CPGs: Clinical practice guidelines
OR: Odds ratio
95% CI: 95% confidence interval
BMI: Body Mass Index
*N*: Number
SD: Standard deviation
NRS: Numeric rating scale
Q2: Second quartile
Q1: First quartile
Q3: Third quartile

## References

[1] Kelly P, Kahlmeier S, Götschi T, Orsini N, Richards J, Roberts N (2014). Systematic review and meta-analysis of reduction in all-cause mortality from walking and cycling and shape of dose response relationship. Int J Behav Nutr Phys Act.

[2] Wojan TR, Hamrick KS (2015). Can walking or biking to work really make a difference? Compact development, observed commuter choice and Body Mass Index. PLoS ONE.

[3] Callaghan MJ (2005). Lower body problems and injury in cycling. J Bodyw Mov Ther.

[4] Silberman MR (2013). Bicycling injuries. Curr Sports Med Rep.

[5] Dingwell JB, Joubert JE, Diefenthaeler F, Trinity JD (2008). Changes in muscle activity and kinematics of highly trained cyclists during fatigue. IEEE Trans Biomed Eng.

[6] Schwellnus MP, Derman EW (2005). Common injuries in cycling: prevention, diagnosis and management. S Afr Fam Pract.

[7] Oggiano L, Leirdal S, Sætran L, Ettema G, Estivalet M (2008). Aerodynamic optimization and energy saving of cycling postures for international elite level cyclists (P114). Eng Sport.

[8] Burnett AF, Cornelius MW, Dankaerts W, O’Sullivan PB (2004). Spinal kinematics and trunk muscle activity in cyclists: a comparison between healthy controls and non-specific chronic low back pain subjects—a pilot investigation. Man Ther.

[9] Silberman MR, Webner D, Collina S, Shiple BJ (2005). Road bicycle fit. Clin J Sport Med.

[10] Jeukendrup AE, Martin J (2001). Improving cycling performance: how should we spend our time and money. Sport Med.

[11] Schwellnus MP, Derman EW, Derman W (2014). Common injuries in cycling: prevention, diagnosis and management. South Afr Fam Pract.

[12] Lane J, Cuthbert R (2017). The prevalence of non-traumatic musculoskeletal injuries in non-professional road cyclists. Physiotherapy.

[13] Wilber CA, Holland GJ, Madison RE, Loy SF (1995). An epidemiological analysis of overuse injuries among recreational cyclists. Int J Sports Med.

[14] Dannenberg AL, Needle S, Mullady D, Kolodner KB (1996). Predictors of injury among 1638 riders in a recreational long-distance bicycle tour: Cycle Across Maryland. Am J Sports Med.

[15] Weiss BD (1985). Nontraumatic injuries in amateur long distance bicyclists. Am J Sports Med.

[16] de Leeuw D, Hox JDD (2008). International handbook of survey methodology (European Association of Methodology Series).

[17] Von Elm E, Altman D, Egger M, Pocock S, Gøtzsche P, Vandenbroucke J (2007). The Strengthening the Reporting of Observational Studies in Epidemiology (STROBE) statement: guidelines for reporting observational studies. Ann Intern Med.

[18] Dionne CE, Dunn KM, Croft PR, Nachemson AL, Buchbinder R, Walker BF (2008). A consensus approach toward the standardization of back pain definitions for use in prevalence studies. Spine (Phila Pa 1976).

[19] Chou R, Qaseem A, Snow V, Casey D, Cross J, Shekelle P (2007). Diagnosis and treatment of low back pain: a joint clinical practice guideline from the American College of Physicians and the American Pain Society. Ann Intern Med.

[20] World Health Organization (2013). Priority medicines for Europe and the world 2013 update, Chap. 6.24.

[21] Ganesan S, Acharya AS, Chauhan R, Acharya S (2017). Prevalence and risk factors for low back pain in 1,355 Young adults: a cross-sectional study. Asian Spine J.

[22] Andersson GB (1999). Epidemiological features of chronic low-back pain. Lancet (London, England).

[23] Maselli F, Storari L, Barbari V, Colombi A, Turolla A, Gianola S (2020). Prevalence and incidence of low back pain among runners: a systematic review. BMC Musculoskelet Disord.

[24] Shiri R, Coggon D, Falah-Hassani K (2018). Exercise for the prevention of low back pain: systematic review and meta-analysis of controlled trials. Am J Epidemiol.

[25] Alzahrani H, Mackey M, Stamatakis E, Zadro JR, Shirley D (2019). The association between physical activity and low back pain: a systematic review and meta-analysis of observational studies. Sci Rep.

[26] Mok LC, Lee IFK (2008). Anxiety, depression and pain intensity in patients with low back pain who are admitted to acute care hospitals. J Clin Nurs.

[27] Stafford MA, Peng P, Hill DA (2007). Sciatica: a review of history, epidemiology, pathogenesis, and the role of epidural steroid injection in management. BJA Br J Anaesth.

[28] Clarsen B, Krosshaug T, Bahr R (2010). Overuse injuries in professional road cyclists. Am J Sports Med.

[29] Maselli F, Ciuro A, Mastrosimone R, Cannone M, Nicoli P, Signori A (2015). Low back pain among Italian rowers: a cross-sectional survey. J Back Musculoskelet Rehabil.

[30] Streisfeld GM, Bartoszek C, Creran E, Inge B, McShane MD, Johnston T (2017). Relationship between body positioning, muscle activity, and spinal kinematics in cyclists with and without low back pain: a systematic review. Sports Health.

[31] Trompeter K, Fett D, Platen P (2017). Prevalence of back pain in sports: a systematic review of the literature. Sport Med.

[32] Korff T, Romer L, Mayhew I, Martin J (2007). Effect of pedaling technique on mechanical effectiveness and efficiency in cyclists. Med Sci Sports Exerc.

[33] Galindo-Martínez A, López-Valenciano A, Albaladejo-García C, Vallés-González JM, Elvira JLL (2021). Changes in the trunk and lower extremity kinematics due to fatigue can predispose to chronic injuries in cycling. Int J Environ Res Public Health.

[34] Harries SK, Lubans DR, Callister R (2015). Systematic review and meta-analysis of linear and undulating periodized resistance training programs on muscular strength. J Strength Cond Res.

[35] Kristensen J, Franklyn-Miller A (2012). Resistance training in musculoskeletal rehabilitation: a systematic review. Br J Sports Med.

[36] Negrini S, Giovannoni S, Minozzi S, Barneschi G, Bonaiuti D, Bussotti A (2006). Diagnostic therapeutic flow-charts for low back pain patients: the Italian clinical guidelines. Eura Medicophys.

[37] Battista S, Salvioli S, Millotti S, Testa M, Dell’Isola A (2021). Italian physiotherapists’ knowledge of and adherence to osteoarthritis clinical practice guidelines: a cross-sectional study. BMC Musculoskelet Disord.

[38] Ivanova JI, Birnbaum HG, Schiller M, Kantor E, Johnstone BM, Swindle RW (2011). Real-world practice patterns, health-care utilization, and costs in patients with low back pain: The long road to guideline-concordant care. Spine J.

[39] Harle CA, Danielson EC, Derman W, Stuart M, Dvorak J, Smith L (2018). Analgesic management of pain in elite athletes: a systematic review. Clin J Sport Med.

[40] Outram SM, Stewart B (2015). Condemning and condoning: elite amateur cyclists’ perspectives on drug use and professional cycling. Int J Drug Policy.

[41] Pillastrini P, Gardenghi I, Bonetti F, Capra F, Guccione A, Mugnai R (2012). An updated overview of clinical guidelines for chronic low back pain management in primary care. Joint Bone Spine.

[42] Koes BW, Van Tulder M, Lin CWC, Macedo LG, McAuley J, Maher C (2010). An updated overview of clinical guidelines for the management of non-specific low back pain in primary care. Eur Spine J.

[43] Carville S, Constanti M, Kosky N, Stannard C, Wilkinson C (2021). Chronic pain (primary and secondary) in over 16s: summary of NICE guidance. BMJ.

[44] Smeets R, Köke A, Lin CW, Ferreira M, Demoulin C (2011). Measures of function in low back pain/disorders: Low Back Pain Rating Scale (LBPRS), Oswestry Disability Index (ODI), Progressive Isoinertial Lifting Evaluation (PILE), Quebec Back Pain Disability Scale (QBPDS), and Roland-Morris Disability Questionnaire. Arthritis Care Res.

[45] Monticone M, Ferrante S, Maggioni S, Grenat G, Checchia GA, Testa M (2014). Reliability, validity and responsiveness of the cross-culturally adapted Italian version of the core outcome measures index (COMI) for the neck. Eur Spine J.

[46] Robinson J, Cook J, Purdam C, Visentini P, Ross J, Maffulli N (2001). The VISA-A questionnaire: a valid and reliable index of the clinical severity of Achilles tendinopathy. Br J Sports Med.

