# Supplementary material for: Prevalence, Characteristics, Association Factors of and Management Strategies for Low Back Pain Among Italian Amateur Cyclists: an Observational Cross-Sectional Study
Source: Sports Med Open. 2021 Oct 28;7:78. doi: 10.1186/s40798-021-00370-2 (PMC8555071; doi:10.1186/s40798-021-00370-2)
Supplement: Supplementary file 1 — Additional file 1. Translated version of the questionnaire provided to Italian amateur cyclists. [file 40798_2021_370_MOESM1_ESM.docx]

**Appendix A: Translated version of the questionnaire †**

**Prevalence, Characteristics, Associations Factors of and Management Strategies for Low Back Pain among Italian Amateur Cyclists: an Observational Cross-sectional Study**

**Springer – Sports Medicine Open**

Simone Battista^1^, Lucia Grazia Sansone^1^, Marco Testa*^1^.

1. Department of Neurosciences, Rehabilitation, Ophthalmology, Genetics, Maternal and Child Health, University of Genova, Campus of Savona, Italy

**†** Please, note that the ethics committee documents, signed by the participants for the informed consent, privacy and anonymous data publication are not here reported and that they were inserted at the very beginning of the questionnaire.

*Address all correspondence to Marco Testa at [marco.testa@unige.it](mailto:marco.testa@unige.it), Department of Neurosciences, Rehabilitation, Ophthalmology, Genetics, Maternal and Child Health, University of Genova, Campus of Savona, Via Magliotto 2, 17100, Savona, SV, Italy

Exclusion criteria

1. Have you ever had vertebral fractures or back surgery (e.g., vertebral stabilisation)?

- Yes [end of the survey]
- No

1. Do you have severe spondylolisthesis (3rd degree or greater) spondylolisthesis?

- Yes [end of the survey]
- No

1. Do you have rheumatic diseases?

- Yes [end of the survey]
- No

Demographic data

1. Sex

- Male
- Female

1. Age (years)
2. Height [cm]
3. Weight [kg]
4. Do you smoke?

- Yes
- No

1. How many hours do you sleep per night?

- Less than 6 h
- 6-7 h
- 7-8 h
- 8-9 h
- More than 9 h

1. Your work-type is:

- Sedentary
- Static
- Dynamic
- Heavy
- Unemployed

1. How long have you been cycling?

- Less than 2 years
- 2-5 years
- More than 5 years

1. You are currently registered as:

- Competitor
- No Competitor
- No Membership

1. How many times do you train on a bicycle in a week?

- Once to twice
- Thrice to four times
- Five to six times
- More than six times

1. How many hours do you train on a bicycle in a week?

- Less than 5 h
- Between 5 and 12 h
- Between 13 and 20 h
- More than 20 h

1. Do you have a coach or do you follow training schedules?

- Yes
- No

1. Do you use tools to evaluate your workouts and your physical shape?

- No one
- Heart rate monitor
- Heart rate and power monitors

1. Do you practice stretching sessions?

- Never
- Rarely
- Once or twice a week
- Often

1. In addition to cycling, do you regularly practice other sports at least once a week?

- Yes
- No

Cycling races

1. How many races do you run every year?

- None [go to question n° 21]
- Between 1 and 4 races
- Between 5 and 8 races
- Between 9 and 12 races
- More than 12 races

1. Which type of races do you participate in most frequently?

- Track cycling
- Cyclosportive
- Hillclimbing

Technical aspects

1. Have you ever performed specific training to improve your pedalling technique (eg pedalling with one leg)?

- Yes
- No

1. Have you ever had a biomechanical visit?

- Yes
- No [go to question n° 25]

1. Why did you have a biomechanical visit?

- Improve performance [go to question n° 25]
- Injury prevention [go to question n° 25]
- Pain-discomfort

1. After the biomechanical visit your pain/discomfort was:

- Unchanged
- Partially improved
- Improved a lot
- Completely disappeared

Back pain in life

25) Have you ever had pain in the areas highlighted in the figure below in your life, such as affect your daily activities (getting dressed, bathing, walking, driving, exercising) or did you change your daily habits for more than one day?


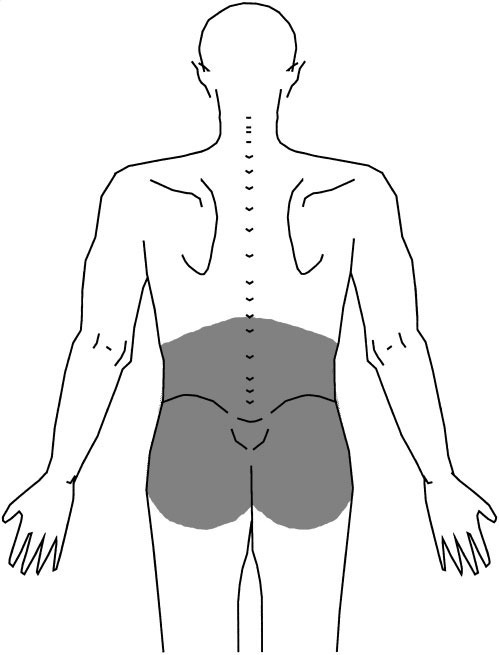


- Yes
- No [go to question n° 39]

1. How many times in your life have you had back pain episodes that required you to change your daily habits and activities for more than one day? (only integer numbers from 1 to 99 are accepted)
2. Have you ever had pain radiated to the lower limb?

- Yes
- No [go to the question n° 30]

1. Did you have pain radiating to the lower limb

- To the knee
- Beneath the knee

1. Did you have any other symptoms in the lower limb? (multiple choice is allowed)

- No
- Tingling
- Burning
- Strength loss
- Sensitivity loss

Back pain in the past 12 months

1. In the past 12 months have you ever had pain in the areas highlighted in the figure below, such as affect your daily activities (getting dressed, bathing, walking, driving, exercising) or having changed your daily habits for more than one day?


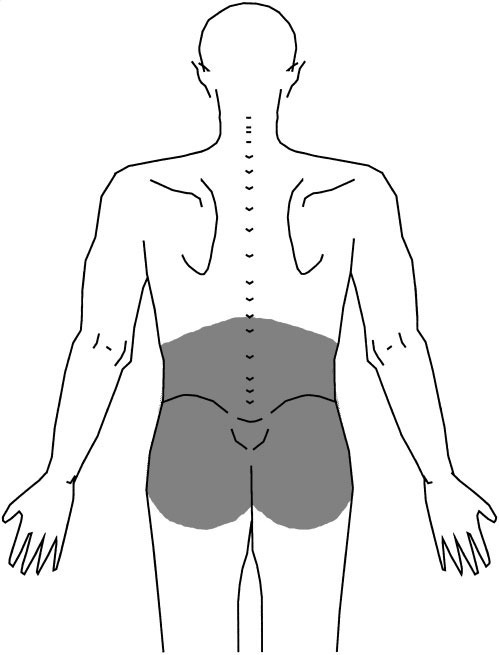


- Yes
- No [go to question n° 34]

1. How many times in the past 12 months have you had back pain episodes that have required you to change your daily habits and activities for more than one day? (only integers numbers between 1 and 99 are accepted)

Back pain within the past 4 weeks

1. In the last 4 weeks have you ever had pain in the areas highlighted in the figure below, such as to affect your daily activities (dressing, bathing, walking, driving, exercising) or to change your daily habits for more than a day?


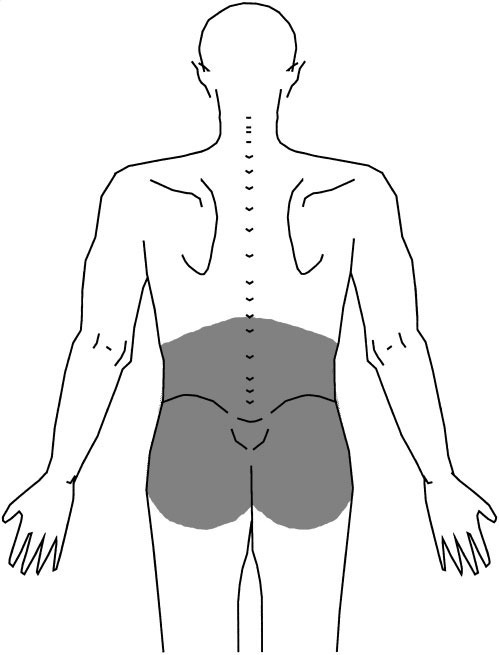


- Yes
- No [go to question n ° 34]

1. How many times in the past 4 weeks have you had back pain episodes that required to change your daily habits and activities for more than one day? (only integers numbers between 1 and 99 are accepted)

Last episode of back pain

1. In reference to the last episode of back pain that required you to change your habits and daily activities for more than one day, how long did the pain last?

- Some days
- One month
- Three months
- More than three months

1. How intense was the pain on a scale from 0 to 10, where 0 means “no pain” and 10 “the Worst imaginable pain"? [slider from 0 to 10]
2. Did you have pain radiating to the lower limb?
3. Have you noticed if back pain events coincided with a particular period of stress (school, work, family)?

- Yes
- No

Back pain and cycling

1. Have you ever had back pain before starting road cycling?

- Yes
- No

Back pain on a bicycle

1. Have you ever had back pain during or after cycling?

- Yes
- No [end of the survey]

1. How has back pain affected your cycling workouts?

- I have continued to train normally despite the pain [go to question n° 42]
- I had to reduce the intensity and frequency of training [go to question n° 42]
- I had to change the type of training [go to question n° 42]
- I had to stop training

1. For how many days did you suspend training?
2. When did back pain occur?

- Only during the bike ride
- Both during and after the bike ride
- Only after the bike ride [go to question n° 43 and then go to question n° 46]
- Always

1. On average how badwas the pain on a scale of 0 to 10, where 0 means “no pain” e 10 is “the worst pain imaginable”? [slider from 0 to 10]
2. Pain during cycling occurred:

- Only under exertion
- After a few hours
- Independently from the effort
- Right away
- Regardless of the effort

1. What did you usually do to reduce back pain while cycling?

- I flexed my back (hunching it)
- I extended my back (arching it, flattening it, straightening it)
- Pedalling more agile
- I pedalled harder
- I reduced the speed and intensity of the effort
- I stopped e I got off the bike
- None of this

1. ​​Have you noticed if the back pain coincided with a particular period?

- Training period
- Pre-competition period
- Competition period [go to question n° 48]
- Unloading period [go to question n° 48]
- No particular period [go to question n° 48]

1. What was the training mainly characterised by?

- Aerobic fund in the plains
- Works by strength (SFR)
- Short high intensity repetitions (2-5 min)
- Long high intensity repetition (10-15 min)
- Long climbs in threshold (20-40 min)
- Long climbs at low intensity (> 30min)
- Sprints (20-60 sec)

1. Have you noticed if the back pain coincided with a change in the asset of the bicycle (Saddle height or back, Saddle-handlebar distance or difference, Position of the cleats) or as a change of some components (saddle, handlebar, pedals)?

- Yes
- No

1. Do you think that cycling affects your back pain?

- Cycling does not affect my back pain
- Cycling negatively affects my pain back
- Cycling has a positive effect on my back pain

1. Do you think cycling is the cause of your back pain?

- Yes
- No

1. Have you ever contacted a healthcare professional for your back pain?

- Yes
- No [go to question n° 53]

1. To solve your back pain, which professional did you turn to? (multiple choice allowed)

- Doctor
- Physiotherapist
- Osteopath
- Chiropractor
- Masseur
- Biomechanical operator
- Other (please specify)

1. Have you ever taken medication(s) to reduce symptoms?

- Yes
- No [go to the question n° 56]

1. What medication(s) did you take to reduce your symptoms?

- Cortisone
- Non-anti-inflammatory drugs steroids (eg. Voltaren, Dicloreum, Aspirin, Moment, Brufen, Aulin, Feldene)
- Painkillers (eg. Tachipirina, Tachidol, Momendol)

1. Who did recommend those medications to you?

- Doctor
- Friends or family
- None

1. Do you practice bodyweight or gym exercises to strengthen your back?

- Never
- Rarely
- Only in certain periods of the year
- 1-2 times a week
- Almost every day
